# Recent Advances in Neuroimaging Methods

**DOI:** 10.1155/2008/218582

**Published:** 2008-05-12

**Authors:** Oury Monchi, Habib Benali, Julien Doyon, Antonio P. Strafella

**Affiliations:** ^1^Functional Neuroimaging Unit, Montreal Geriatric's Institute, University of Montreal, Montreal, QC, Canada H3W 1W5; ^2^Departement of Radiology, University of Montreal, Montreal, QC, Canada H3T 1J4; ^3^Laboratoire d'Imagerie Fonctionnelle (UMR S678), INSERM-UPMC, CHU-Pitié-Salpêtrière, 75013 Paris, France; ^4^Departement of Psychology, University of Montreal, Montreal, QC, Canada H2V 2S9; ^5^Toronto Western Hospital and CAMH-PET Centre, University of Toronto, Toronto, ON, Canada M5T 1R81

Over the past decade, we have seen a large explosion of anatomical and functional neuroimaging techniques, allowing exciting investigation of new aspects of the human brain functions with respect to cognition, learning, and memory. These developments have occurred with respect to the machinery (diverse methods of acquisition), the methods for analysis, and the extent of clinical applications. This issue gives representative examples of these three avenues of development.

Recent advances in magnetic resonance imaging acquisition techniques have not only focused on functional sequences but also on anatomical ones. One important contribution to this field has been the recent development of diffusion weighted imaging magnetic resonance (MR) sequences in order to study the quality of white matter connectivity and importantly perform tractography in order to study for the first time anatomical connectivity in the human brain in vivo. Indeed, five articles in the present issue focus on such methods. Leh et al. use diffusion tensor imaging (DTI) tractography to study cortical and subcortical connectivity of the pulvinar in the living human brain. Their results are in accordance with those previously observed in monkeys and provide further support for an important role of the pulvinar in human visual information processing and spatial attention. One difficulty often encountered in performing analyses of effective connectivity (such as structural equations modeling or dynamical causal modeling) of fMRI data is that one usually has to provide an initial network model to be validated. This model should ideally be based on known anatomical connections between brain regions. However, such information in humans is still incomplete. In their article, Fonteijn et al. present a study that revisits existing functional networks using DTI. The obtained results are of great interest for all researchers in the field. Information is provided about the likelihood of region connections via tractography. For a number of connections the authors find anatomical connectivity that corresponds to the proposed functional paths. They provide compelling examples showing that the use of DTI tractography is a valuable tool to define the anatomical networks required to perform good analysis of effective connectivity. From a more methodological viewpoint, Jbabdi et al. propose a model for the structure of the brain which considers anatomical connections between various cerebral regions as being geodesic with respect to a metric given by the inverse of the diffusion tensor. Using this geodesic method, they are able to construct a path connecting every pair of brain regions. However, the brain is obviously not fully connected, and consequently, they needed to determine whether a given geodesic really represents a white matter fiber tract or not. This is why they also propose an index for the probability of being a fiber, which combines a term that represents the data fit and another term that represents the data confidence. Their new algorithm is tested on simulated data and proves to be computationally fast as well as robust to local perturbation induced by fiber crossing. They also use it on real data to show its feasibility.

A new approach in studying diffusivity in the human brain has been the use of high angular resolution diffusion imaging (HARDI) as an alternative to DTI to overcome the latter method's limitation in complex fiber regions with crossing. Two articles propose novel methods to make the best use of such acquisition sequences. Probabilistic algorithms have been preferred to standard streamline techniques because they are robust to noise in the orientation distribution functions (ODFs) maps and because they can go through bundle crossings that are likely to happen given the effective resolution of the voxels. Perrin et al. develop a new probabilistic algorithm based on the fiber ODF using a Monte Carlo random walk algorithm. Monte Carlo particles move inside the continuous field of q-ball diffusion ODF and are subject to a trajectory regularization scheme. Their new algorithm is tested on simulated data. Segmentation of white matter and subcortical structures from diffusion weighted magnetic resonance imaging, either DTI or HARDI, is fairly recent. Wassermann et al. use a region-based statistical active contour technique on these images of ODFs to find coherent white matter fiber bundles and a nonlinear spectral-clustering algorithm is presented in order to segment different fiber bundles.

The use of transcranial magnetic stimulation (TMS) has greatly increased during the last five years. While not being an imaging method per se, it does allow to perform “brain mapping” by studying change in behavior and performance when stimulating a specific cortical region. In combination with other brain imaging methods such as Positron emisson tomography or functional MRI, it also allows for the study of functional connectivity. Finally, it is also being explored as a therapeutic tool for patients with neurological and psychiatric disorders. In their manuscript, Ko et al. applied rTMS to right dorsolateral prefrontal cortex (DLPFC) and the vertex during different temporal phases of the Wisconsin card sorting task (WCST), an extensively used neuropsychological task to assess executive processes. Performance on the WCST specifically deteriorated when applying rTMS to the DLPFC (and not the vertex) when it was applied during the feedback period (when the participant plans the next response) but not when it was applied during the matching period (when the response is executed).  This selective impairment of the DLPFC is consistent with its proposed role in monitoring events in working memory. Rektorova et al., for their part, investigated whether rTMS can induce beneficial effects on L-Dopa induced dyskinesias in Parkinson’s disease. Their preliminary results indicate that rTMS of the DLPFC in these patients does have an improving effect on dyskinesias possibly by inducing a depression of motor cortex excitability, while stimulating the motor cortex directly does not provide the same effect.

One issue of great debate regarding blood oxygenated level dependant (BOLD) functional MRI is its relationship to cerebral blood flow and metabolism under different conditions. Previous studies have suggested that during selective activation of a subset of the zones comprising a columnar system in the visual cortex, perfusion increases uniformly in all columns in the system, while an increase in oxidative metabolism occurs predominantly in the activated column. If this were true, one would expect a disproportionally large BOLD increase compared with blood flow, for a highly localized response in the cortex as opposed to a more diffuse one. To address this issue, Gauthier and Hoge used arterial spin labeling in a group of young adults while performing a monocular and a binocular task. Their results show that the ratio of BOLD to cerebral blood flow effects do not differ significantly between the two stimulation conditions, indicating comparable coupling between flow and oxidative metabolism in V1, regardless of the columnar fraction that was activated.

The investigation of the patterns of connectivity in largescale extended brain networks in the context of BOLD fMRI is a complex task that has also been the source of much attention during the last few years. In their article, Perlbarg and Marrelec review the methods used so far, discuss some of the issues that have to be faced, and propose some avenues for more efficient exploration of such networks. They describe the early correlation approaches that have been used, socalled functional connectivity studies, but point out that the exploration of a whole network would require the successive computations of many functional connectivity maps, each map being used for the selection of the seed voxel, which does not prove very realistic. They also advocate that most methods developed so far for effective connectivity have been of restricted use for studying extended large-scale networks, as their intrinsic complexity prevents them from modeling systems with that many degrees of freedom. They propose that mathematical methods coming from graph and/or network theory may be well suited to deal with such problems, and stress the importance of comparing results coming from other imaging modalities to validate and better understand the large-scale network approach in fMRI.

Multiple methods have been developed recently to perform meta-analysis of functional neuroimaging data. Many of them have been task dependent. Peiffer et al. propose to extend to BOLD fMRI, a method that has been proposed in the mid-60’s to assess the relationship between response times in young and older adults across a variety of tasks called the Brinley plots. In the proposed method, a linear regression is performed over the average BOLD activity map taken over different scanning sessions (while performing different tasks) taken from each group to be compared (e.g., older versus younger adults). This provides a relatively easy method to perform a meta-analysis to evaluate two different groups that take into account between-task differences.

Finally, on the clinical side, Bernad and Doyon review the role of noninvasive neuroimaging techniques such as fMRI and TMS in understanding how the neural connections are altered as a consequence to cerebrovascular injury, the neural mechanisms that underlie neurorehabilitation in stroke, as well as motor memory consolidation in healthy adults. They argue that these methods have the potential to be used as clinical tools to promote and optimize individualized motor recovery in stroke patients.

Altogether, these papers constitute a representative sample of the state of the art in neuroimaging methodology and we hope they will be of great interest to a large number of scientists and clinicians in the field.

